# Circulating FGF21 and Ketone Bodies Modify the Risk of MASLD and Mortality: Insights from the PREVEND Cohort Study

**DOI:** 10.3390/ijms26115059

**Published:** 2025-05-24

**Authors:** Mateo Chvatal-Medina, Yakun Li, Wendy A. Dam, Margery A. Connelly, Han Moshage, Stephan J. L. Bakker, Robin P. F. Dullaart, Adrian Post

**Affiliations:** 1 Department of Gastroenterology and Hepatology, University Medical Center Groningen, University of Groningen, P.O. Box 30.001, 9700 RB Groningen, The Netherlands; m.chvatal-medina@umcg.nl (M.C.-M.); y.li01@umcg.nl (Y.L.); a.j.moshage@umcg.nl (H.M.); 2 Department of Internal Medicine, University Medical Center Groningen, University of Groningen, P.O. Box 30.001, 9700 RB Groningen, The Netherlands; w.a.dam01@umcg.nl (W.A.D.); s.j.l.bakker@umcg.nl (S.J.L.B.); a.post01@umcg.nl (A.P.); 3 Labcorp, 100 Perimeter Park, Morrisville, NC 27560, USA; connem5@labcorp.com

**Keywords:** fibroblast growth factor 21 (FGF21), ketone bodies, metabolic dysfunction-associated steatotic liver disease (MASLD), mortality, Prevention of Renal and Vascular End-stage Disease (PREVEND) cohort study

## Abstract

Fibroblast growth factor 21 (FGF21) and ketone bodies are markers of metabolic dysregulation, independently associated with metabolic-dysfunction-associated steatotic liver disease (MASLD) and mortality. We studied their interaction with MASLD and all-cause mortality in 6025 participants from the Prevention of Renal and Vascular End-stage Disease (PREVEND) cohort. Plasma FGF21 (immunoassay) and ketone body concentrations (nuclear magnetic resonance spectroscopy) were measured at baseline. A Fatty Liver Index ≥60 was used as a proxy of MASLD. Logistic regression assessed associations with MASLD, and Cox models evaluated all-cause mortality over a median follow-up of 10.3 years. FGF21 and ketone bodies were not correlated (r = 0.02, *p* = 0.06), but FGF21 (OR: 1.93 [1.81–2.05], *p* < 0.001) and ketone bodies (OR: 1.29 [1.19–2.05], *p* < 0.001) were independent of each other associated with MASLD, with a positive interaction (*p* = 0.004). Higher FGF21 (HR: 1.24, 95% CI: 1.16–1.32, *p* < 0.001) and ketone bodies (HR: 1.46, 95% CI: 1.34–1.59, *p* < 0.001) were associated with mortality, as well as with a positive interaction (*p* = 0.038). After adjustment for potential confounders, only ketone bodies remained independently associated, while the association of FGF21 became dependent on ketone body levels (interaction *p* = 0.005). These biomarkers may serve as integrated metabolic stress markers, improving risk stratification for MASLD and adverse outcomes.

## 1. Introduction

Fibroblast growth factor 21 (FGF21) and ketone bodies are each involved in metabolic adaptation, particularly in response to nutrient deprivation. However, alterations in these markers are also indicators of metabolic dysfunction, which has been increasingly recognized in the context of cardiovascular, kidney, and metabolic diseases [1,2,3]. FGF21 is a stress-inducible hormone, upregulated in conditions of metabolic demand such as fasting and exercise, to facilitate energy mobilization from lipids [4,5]. It promotes fatty acid oxidation, ketogenesis, and glucose regulation, while suppressing hepatic lipogenesis [6,7,8]. While short-term elevations in FGF21 are typically adaptive, persistently elevated levels of FGF21 have been associated with states of metabolic dysfunction such as type 2 diabetes (T2D) and possibly also be associated with metabolic-dysfunction-associated steatotic liver disease/metabolic-dysfunction-associated steatohepatitis (MASLD/MASH) [2,9,10,11,12].

Ketone bodies, namely, β-hydroxybutyrate (BHB), acetoacetate (AcAc), and acetone, are lipid-derived molecules produced by hepatic mitochondria through ketogenesis. Their synthesis is mainly regulated by insulin and free fatty acid availability, and they often serve as an alternative energy supply in conditions of metabolic stress. For example, ketone body elevations may act as a compensatory mechanism to provide energy for the failing heart [13]. Ketone bodies are elevated in acute myocardial infarction and predict larger infarction size [14]. Elevated β-hydroxybutyrate (BHB), the most abundant ketone body, is associated with incident heart failure [15].

FGF21 and ketone metabolism are interconnected since FGF21 enhances ketogenesis by upregulating mitochondrial fatty acid oxidation and activating peroxisome-proliferator-activated receptor alpha (PPARα) in hepatocytes, while ketones regulate FGF21 expression through a feedback loop [2,5]. In the clinical context, both higher FGF21 and higher ketone body levels are independently associated with an increased risk of mortality, but their combined role remains unclear [11,16,17]. Given their shared involvement in energy metabolism, stress adaptation, and lipid homeostasis, FGF21 and ketone bodies may interact to influence disease risk in metabolic conditions such as MASLD. Understanding this relationship could provide insight into mechanisms linking altered energy metabolism to adverse health outcomes.

This study was carried out in the population-based Prevention of Renal and Vascular End-stage Disease (PREVEND) cohort and was designed to investigate the interaction between FGF21 and ketone bodies in relation to MASLD and all-cause mortality.

## 2. Results

### 2.1. Baseline Characteristics of the Study Population

**Figure 1:**
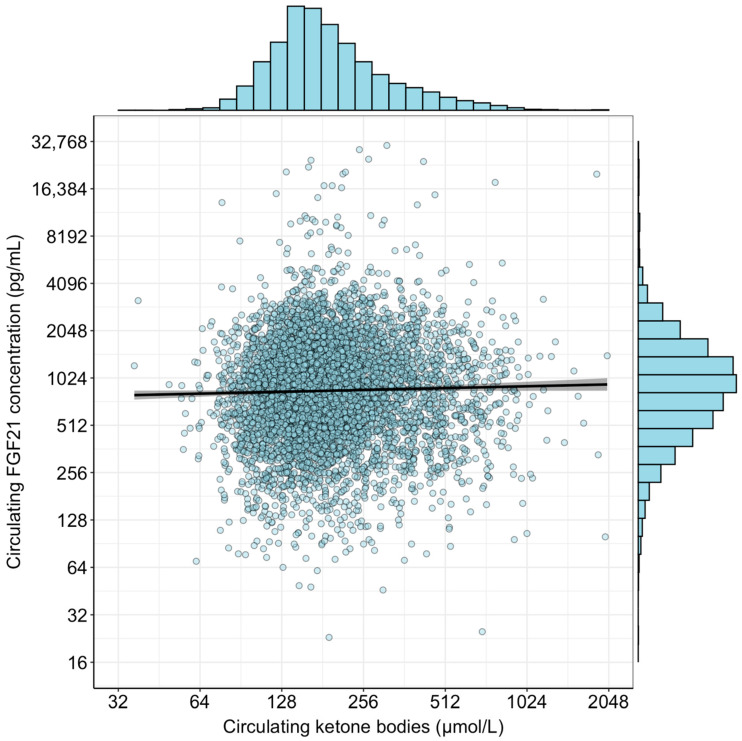
Scatterplot of circulating FGF21 concentration and circulating ketone bodies. Histograms are included to demonstrate the distribution of the variables after log_2_-transformation. No correlation was observed between the two variables (Pearson r = 0.02; *p* = 0.06; R^2^ < 0.001).

A total of 6025 participants were included for these analyses (flowchart in Figure S1). An overview of all baseline characteristics is shown in Table 1. In short, the mean age was 53 ± 12 years, and 49% were men. Median waist circumference was 91 [82–100] cm, mean eGFR was 97 ± 17 mL/min/1.73 m^2^, and median albumin excretion was 9 [6–15] mg/day. Median FGF21 concentration was 888 [534–1374] pg/mL, and median circulating ketone body concentrations were 176 [139–247] µmol/L. A scatterplot of circulating FGF21 concentration and circulating ketone bodies is shown in Figure 1. There was no correlation between circulating FGF21 concentration and circulating ketone bodies (Pearson r = 0.02; *p* = 0.06; R^2^ < 0.001).

### 2.2. Logistic Regression Analyses and Interaction Between FGF21 and Ketone Bodies in MASLD

The median Fatty Liver Index (FLI) was 36 [14–67], and a total of 1864 (31%) had a FLI ≥ 60, as a proxy of MASLD. In participants with a FLI ≥ 60, median FGF21 was 1188 pg/mL and median ketone bodies were 193 µmol/L, whereas in participants with a FLI < 60, median FGF21 was 758 pg/mL and median ketone bodies were 169 µmol/L (Table S1). The BHB/AcAc, ratio was not significantly different between participants with a FLI ≥ 60 and those with FLI < 60 (*p* = 0.15) (Table S1). Logistic regression analyses of circulating FGF21 concentration and circulating ketone bodies with MASLD are shown in Table 2. These analyses demonstrated that both circulating FGF21 concentrations and circulating ketone bodies were independent of each other, and independent of potential confounders associated with MASLD. In the fully adjusted model, higher circulating FGF21 concentration (OR 1.82, 95% CI: 1.64–2.02; *p* < 0.001) and higher circulating ketone bodies (OR 1.19, 95% CI: 1.03–1.38; *p* = 0.016) were associated with a higher prevalence of MASLD. In all tested models, there was a significant positive interaction between circulating FGF21 concentration and circulating ketone bodies, indicating that the association of each variable with MASLD becomes stronger when the levels of both are high. Sensitivity analyses were done with a different FLI cut-off for females to improve its validity as a surrogate for MASLD (Table S2). These showed that despite a change in cut-off value for females, the association persisted between both circulating FGF21 concentrations and circulating ketone bodies with the odds of MASLD. The positive interaction between both markers also persisted. Further sensitivity analyses showed that after stratifying by sex, the association between FGF21 concentration and circulating ketone bodies with MASLD was significant among males and females separately (Table S3).

To demonstrate the effect of this interaction in tabular format, we performed stratified analyses across the median value of ketone bodies for FGF21 and the median value of FGF21 for ketone bodies (Table 3). These analyses showed that the association of circulating FGF21 concentration with MASLD was weaker in participants with lower ketone bodies (OR per doubling of FGF21: 1.58, 95% CI: 1.35–1.85; *p* < 0.001) as compared to participants with higher ketone bodies (OR per doubling of FGF21: 2.05, 95% CI: 1.78–2.37; *p* < 0.001) (difference across strata: *p* = 0.02). Similarly, the association of circulating ketone bodies with MASLD was weaker in participants with lower FGF21 concentration (OR per doubling of FGF21: 0.98, 95% CI: 0.78–1.23; *p* = 0.86), as compared to participants with higher FGF21 concentration (OR per doubling of FGF21: 1.41, 95% CI: 1.73–1.70; *p* < 0.001) (difference across strata *p* = 0.02). In sensitivity analyses using different FLI cut-off values for females, a similar association was reported for both markers (Table S4). Similar results were found when using the median value at event occurrence (Table S5).

**Figure 2:**
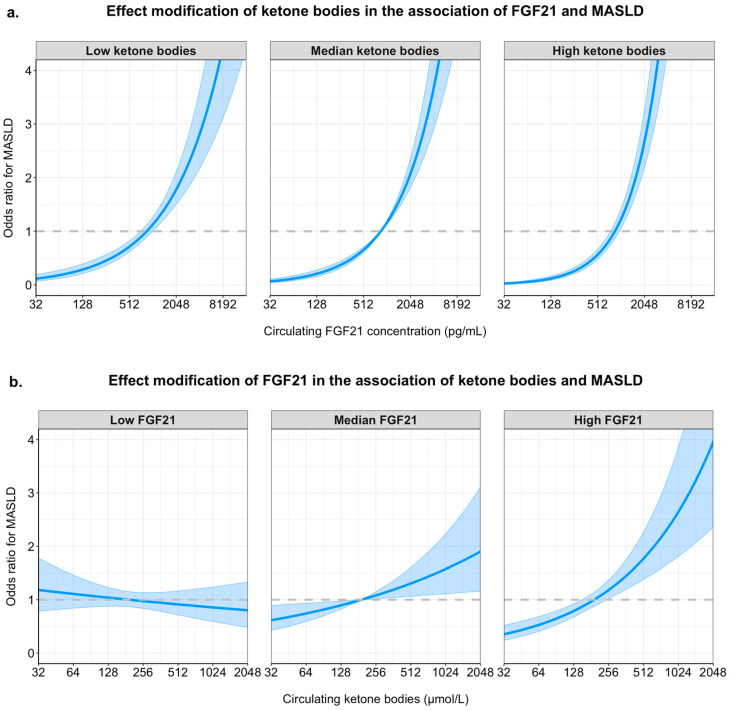
Graphical representation of the interactions between FGF21 concentration and ketone bodies in their associations with MASLD. Panel (**a**) shows the adjusted odds ratios (y-axis) for MASLD across increasing circulating FGF21 concentrations (x-axis, pg/mL, log_2_ scale) for three ketone body levels: 10th percentile (low), median (moderate), and 90th percentile (high). Panel (**b**) shows odds ratios for MASLD across increasing ketone body concentrations (x-axis, µmol/L, log_2_ scale), stratified by low (10th percentile), median, and high (90th percentile) levels of FGF21. All models include both FGF21 and ketone bodies (log_2_-transformed), as well as their interaction term, and are adjusted for age, sex, waist circumference, eGFR, 24-hour urinary albumin excretion, alcohol intake, smoking status, HDL cholesterol, total cholesterol, systolic blood pressure, and diabetes status. Shaded bands represent 95% confidence intervals.

To visualize the interaction between circulating FGF21 concentration and ketone bodies in the fully adjusted model, the association of FGF21 concentration with MASLD is plotted for the 10th percentile, the median value, and the 90th percentile of ketone bodies in the upper panel of Figure 2. Likewise, the association of ketone bodies with MASLD is plotted for the 10th percentile, the median value, and the 90th percentile of FGF21 concentration in the lower panel of Figure 2.

### 2.3. Longitudinal Analyses and Interaction of FGF21 and Ketone Bodies on All-Cause Mortality

**Figure 3:**
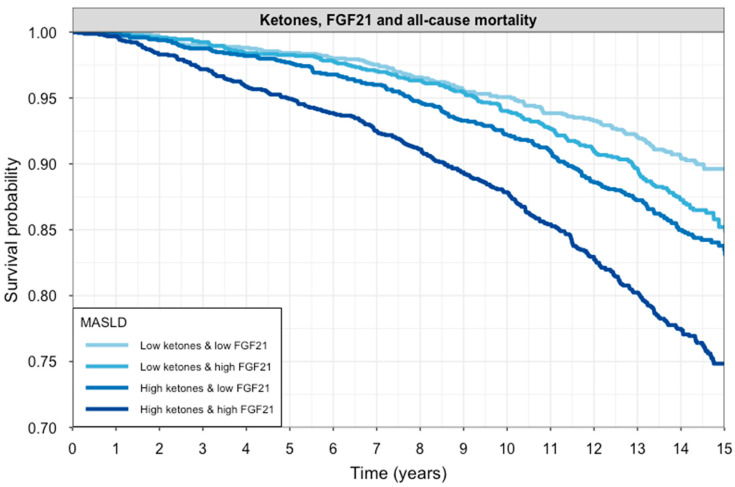
Kaplan–Meier curves for all-cause mortality according to circulating FGF21 and ketone body levels. Participants were stratified into four groups based on median splits of FGF21 and ketone bodies. Survival probability over 15 years is shown for each subgroup. The darkest line represents individuals with both high FGF21 and high ketones, while the lightest line represents those with both biomarkers below the median. Groups with elevated FGF21 and/or ketones had significantly lower survival probabilities (log-rank test: *p* < 0.001).

During a median follow-up of 10.3 (8.2; 14.3) years, a total of 843 (14%) participants died. Participants who died had both higher FGF21 concentration (1031 [686–1657] vs. 860 [517–1328] pg/mL) and ketone bodies (205 [157–297] vs. 173 [136–240] µmol/L) as compared to participants who remained alive (both *p* < 0.001). A Kaplan–Meier curve of survival according to FGF21 concentration and ketone bodies stratified as high or low based on the median value is shown in Figure 3 (log-rank test: *p* < 0.001). An overview of Cox regression analyses is shown in Table 4. These analyses demonstrate that both higher FGF21 concentration and higher ketone bodies were associated with a higher risk of all-cause mortality. The associations of FGF21 concentration were significant in models 1 to 3 but lost significance in the fully adjusted model 4, although the interaction term remained significant, implying that the effect on mortality is dependent on the ketone body concentration in this model. The association of ketone bodies with all-cause mortality was significant in all models. In each model, there was a significant and positive interaction term, indicating that the association of each variable with all-cause mortality becomes stronger at higher values of the other variable. Sensitivity analyses also showed significance in the association when stratifying between sexes in most models (Table S6). Further analyses also showed that the association was sustained in most models when stratifying between causes of death, mostly for non-cardiovascular deaths (Table S7).

To demonstrate the effect of this interaction in tabular format, we performed stratified analyses across the median value of ketone bodies for analyses of FGF21 and FGF21 for analyses of ketone bodies (Table 5). These analyses show that the association of FGF21 concentration with all-cause mortality was weaker in participants with lower ketone bodies (HR per doubling of FGF21: 1.03, 95% CI: 0.91–1.16; *p* < 0.001) as compared to participants with higher ketone bodies (HR per doubling of FGF21: 1.11, 95% CI: 1.02–1.21; *p* = 0.22). Similarly, the association of ketone bodies with all-cause mortality was weaker in participants with lower FGF21 concentration (HR per doubling of FGF21: 1.11 95% CI: 0.96–1.28); *p* = 0.17), as compared to participants with higher FGF21 concentration (HR per doubling of FGF21: 1.26, 95% CI: 1.11–1.43; *p* < 0.001).

**Figure 4:**
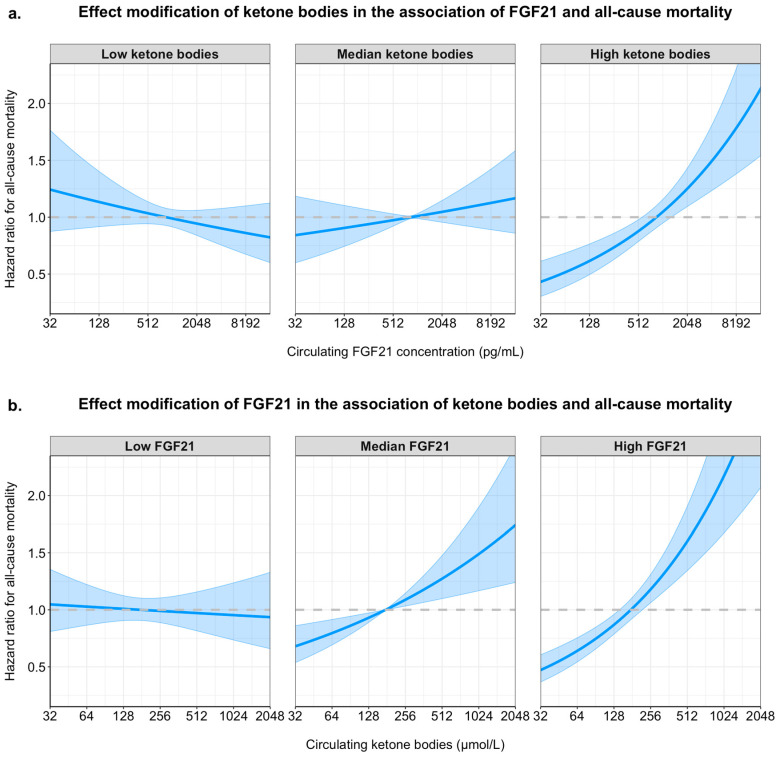
Interaction effects of FGF21 and ketone bodies on all-cause mortality in Cox proportional hazards models. Hazard ratios for all-cause mortality are plotted across the range of circulating FGF21 (panel (**a**)) and ketone bodies (panel (**b**)), modeled with interaction terms and adjusted for age, sex, waist circumference, estimated glomerular filtration rate (eGFR), urinary albumin excretion, alcohol intake, smoking status, total cholesterol, HDL cholesterol, systolic blood pressure, and diabetes status. Panel (**a**) shows the adjusted hazard ratios for FGF21 concentration at different levels of ketone bodies (10th percentile = “low ketone bodies”, median, and 90th percentile = “high ketone bodies”). Panel (**b**) shows the adjusted hazard ratios for ketone body concentration at different levels of FGF21 (10th percentile = “low FGF21”, median, and 90th percentile = “high FGF21”). Shaded areas indicate 95% confidence intervals. Curves are derived from Cox models using log_2_-transformed biomarker values. The interaction shows that the association between one biomarker and mortality is amplified at higher levels of the other.

To visualize the interaction between FGF21 concentration and ketone bodies in the fully adjusted model, the association of FGF21 concentration with all-cause mortality is plotted for the 10th percentile, the median value and the 90th percentile of circulating ketone bodies in the upper panel of Figure 4. Similarly, the association of circulating ketone bodies with the risk of all-cause mortality is plotted for the 10th percentile, the median value and the 90th percentile of circulating FGF21 concentration in the lower panel of Figure 4.

## 3. Discussion

In this study, we investigated the associations between circulating FGF21 concentrations, ketone bodies, and their combined associations with the risks of MASLD and all-cause mortality in a large, population-based cohort. Our results showed that although FGF21 concentration and ketone bodies are not correlated with each other, both higher FGF21 concentrations and higher ketone bodies were independently associated with increased risk of an elevated FLI, as a proxy of MASLD. In addition, their combined elevations demonstrated a significant positive interaction with the risk of overall mortality, suggesting that the association between each variable and MASLD, as well as with risk of mortality, becomes more pronounced as the levels of the other increase. This may imply that simultaneous elevations in both FGF21 and ketone bodies may reflect a state of metabolic stress and dysfunction that amplifies their individual associations with adverse outcomes.

FGF21 and ketone bodies play interrelated roles in metabolic adaptation under conditions of fasting and energy deprivation. FGF21, a member of the FGF family, is a hormone involved in glucose, lipid, and amino acid metabolism and homeostasis, whose importance has been increasingly identified in the context of metabolic stress [18,19]. FGF21 is a stress-inducible hormone that mediates specific glucagon actions and improves insulin sensitivity, which under physiological conditions is mainly secreted by the liver and acts upon target organs including adipose, liver, and muscle tissue in metabolic regulation during fasting and ketogenic states, facilitating energy mobilization from lipids [20,21,22]. However, it is also chronically elevated under metabolic dysfunction, including conditions such as obesity, insulin resistance, T2D, and MASLD [23,24,25]. Despite the favorable metabolic effects observed with exogenous administration of FGF21 analogs such as pegozafermin and zalfermin, which are currently under investigation for MASLD/MASH fibrosis, chronically elevated endogenous FGF21 may paradoxically reflect a state of resistance to FGF21 signaling [26,27]. This resistance might arise due to receptor downregulation and desensitization in response to persistent metabolic stress, which undermines the beneficial metabolic effects of FGF21 and, by contrast, contributes to disease progression [6,28,29]. Hence, and aligned with the findings from our study, it is hypothesized that elevated endogenous FGF21 may indicate a compensatory, yet ineffective, physiological response to chronic metabolic dysfunction.

On the other hand, ketone bodies are lipid-derived metabolites synthesized in the hepatic mitochondria through ketogenesis, which serve as an ancillary energy source during periods of carbohydrate shortage, fasting, or increased energy demand [30,31]. They also serve as signaling molecules for modulation of inflammation, oxidative stress, and epigenetic regulation [32]. However, similar to FGF21, chronically elevated ketone bodies are associated with metabolic stress and disrupted metabolism [32].

Mounting evidence suggests that FGF21 promotes ketone body production by inducing hepatic PPARα signaling, while ketone bodies in turn enhance FGF21 expression, forming a feedback loop that sustains metabolic adaptation in dysfunctional states [33,34]. Prior studies have shown that FGF21 enhances the utilization of ketones by increasing mitochondrial respiratory capacity, partially mediated by AMP-activated protein kinase (AMPK), and FGF21 increases ATP production from β-hydroxybutyrate [34]. This association has been reported in in vitro models such as cardiac muscle cells, where both FGF21 and ketone bodies regulate oxidative stress responses and mitochondrial function through activation of AMPK and nuclear factor erythroid 2-related factor 2 (Nrf2) [35]. Furthermore, FGF21 enhances the ability of neurons to utilize ketone bodies as an energy source, reinforcing its role in metabolic flexibility during periods of scarce glucose availability [34,36]. Overall, the combined dysregulation of FGF21 and ketone bodies may serve as a marker of metabolic stress and progression of disease. Our results build upon this supposition by examining the interaction between these molecules in relation to MASLD and all-cause mortality.

Although FGF21 and ketone bodies are functionally intertwined, and despite their interaction as shown in the present study, several mechanisms might account for the observed lack of correlation. First, there could be a temporal difference between the two, since FGF21 is upregulated transiently and early during fasting but does not continuously rise with ketone body levels [36]. Second, despite the overlapping metabolic pathways, different regulatory inputs from FGF21 and ketone bodies may be partly responsible for the lack of association; for example, FGF21 concentration is influenced by nutrient composition, independent of ketone bodies, while ketone body production is largely dependent on hepatic fatty acid oxidation [35]. Third, the metabolic roles of FGF21 and ketone bodies are different at the tissue level. While FGF21 is secreted by the liver and has endocrine effects on adipose tissue and muscle, ketone bodies are produced in the liver but are rapidly utilized in peripheral tissues. Because circulating ketone body levels reflect both hepatic production and peripheral utilization, they do not necessarily align with FGF21 secretion, and vice versa [30,35].

This study is, to the best of our knowledge, the first to unveil the role of FGF21 and ketone bodies as joint, interacting markers that are significantly associated with MASLD, as well as with overall mortality in a large, population-based cohort. Based on our findings suggesting a synergistic effect and the mechanistic association between FGF21 and ketone bodies, assessing FGF21 and ketone body levels together may provide a comprehensive metabolic risk profile. However, validation in external cohorts is necessary to assess the generalizability of our results and call for several avenues in future research. Future studies should also explore whether the combined elevation could serve as a prognostic marker for MASLD progression and cardiovascular outcomes in patients with MASLD, as well as whether targeted interventions that modify FGF21 signaling or ketone body metabolism could offer therapeutic potential. Finally, in vitro and animal studies should elucidate whether combined elevation of FGF21 and ketone bodies induces maladaptive changes at cellular level.

The noteworthy strengths of this study were the size of the study, the long-term follow-up, and the extensive data collection, allowing for the adjustment of a wide variety of potential confounders. The study has several limitations. First, our observational design precludes causal inference, so the association observed between FGF21, ketone bodies, and clinical outcomes may reflect a shared upstream mechanism, rather than a direct effect. Second, all biomarkers were measured at a single time point, and consequently, we could not capture intra-individual variations through time, and the directionality of the association is unclear, which might leave the possibility of reverse causation in the case of MASLD. Third, although our analyses adjusted for multiple confounders, medications or diets not listed in the variables could modulate these biomarkers. Fourth, there could be an impact from comorbidities not accounted for, such as inflammatory conditions, among others, that leave a residual confounding. Finally, the PREVEND cohort represents mainly white and European individuals, which limits generalizability

## 4. Materials and Methods

### 4.1. Study Design and Participants

This study was conducted within the PREVEND study, a prospective cohort study investigating the prevalence and consequences of microalbuminuria in adults in Groningen, the Netherlands. The study design has been described elsewhere [37].

In brief, between 1997 and 1998, all 85,421 inhabitants aged 28–75 were invited to participate, with 40,856 (47.8%) responding. After exclusions, the PREVEND cohort included 8592 individuals based on urinary albumin concentration.

A second screening (2001–2003) involved 6894 participants and served as the baseline for this study. After excluding 869 participants with missing data on FGF21, ketone bodies, or fatty liver index, 6025 participants remained. Missing covariate data were imputed by predictive mean matching to conserve sample sizes in adjusted regression models. The PREVEND study was approved by the local ethics committee (MEC 96/01/022) and conducted per the Declaration of Helsinki and STROBE guidelines [38]. All participants provided written informed consent. See Figure S1 for the participant flow-chart.

### 4.2. Data Collection

Each screening involved two outpatient visits, three weeks apart. Before the first visit, participants completed self-administered questionnaires on demographics, cardiovascular and renal history, smoking, and medication use. Height and weight were measured without shoes or heavy clothing to calculate body mass index (BMI). Blood pressure was measured on the right arm using a Dinamap XL Model 9300 series device (Johnson & Johnson, New Brunswick, NJ, USA), with values averaged from the last two readings of both visits. Patients were instructed to fast overnight, and baseline EDTA plasma samples were collected between 8:00 and 10:00 a.m., i.e., after a 10 hour fast and stored at −80 °C until analysis.

Plasma FGF21 concentrations were measured using the Mesoscale U-PLEX immunoassay. Intra- and inter-assay precision ranged from 2.5% to 12.1%. Method validation is detailed elsewhere [2].

Total ketone body concentration was calculated as the sum of β-hydroxybutyrate, acetoacetate, and acetone, measured in EDTA-plasma using the Vantera^®^ Clinical Analyzer (Labcorp, Morrisville, NC, USA), a high-throughput 400 MHz 1H nuclear magnetic resonance (NMR) spectroscopy platform. Method validation is detailed elsewhere [12]. Intra- and inter-assay precision coefficients of variation ranged from 1.3 to 9.3% for β-hydroxybutyrate, 3.1 to 7.7% for acetoacetate, and 3.8 to 9.1% for acetone.

Other laboratory measurements were performed in the central laboratory of the University Medical Center Groningen using standard protocols, as described previously [2,11,39,40].

### 4.3. Outcome Data

The outcomes of the current study were the presence of MASLD cross-sectionally and all-cause mortality longitudinally. MASLD was defined according to the updated consensus criteria as hepatic steatosis in the presence of metabolic dysfunction. As liver biopsy was not feasible in this population-based cohort, hepatic steatosis was operationalized using the FLI, a widely validated non-invasive score that incorporates BMI, waist circumference, serum triglycerides, and gamma-glutamyltransferase (GGT) [41,42]. The FLI was calculated as follows:

FLI = (e^(0.953 × loge (triglycerides) + 0.139 × BMI + 0.718 × loge (ggt) + 0.053 × waist circumference − 15.745))/(1 + e^(0.953 × loge (triglycerides) + 0.139 × BMI + 0.718 × loge (ggt) + 0.053 × waist circumference − 15.745)) × 100, where GGT is gamma-glutamyltransferase. The optimal FLI cut-off for detecting suspected MASLD by ultrasonography is 60, with 88% accuracy [43]. Participants with a FLI score ≥ 60 were classified as having MASLD. Sensitivity analyses were performed, modifying the cut-off value between male and female participants, with FLI score ≥ 60 for males and ≥32 for females [44].

The prospective outcome was all-cause mortality, obtained from the municipal register. Follow-up time was defined from the second screening (baseline) until death, loss to follow-up, or 01-01-2017, whichever came first. For individuals who moved to an unknown destination, the date of removal from the municipal registry was used. The cause of death was determined by linking death certificate numbers to primary causes coded by physicians at the Central Bureau of Statistics using the ICD-10 classification.

### 4.4. Statistical Methods

Statistical analyses were performed with R version 4.3.0 (Vienna, Austria) (http://cran.r-project.org/, accessed on 15 October 2024). Results were expressed as mean ± standard deviation (SD), median [interquartile range], or number (percentage) for normally distributed, skewed, and categorical data, respectively. A two-sided *p* value < 0.05 was considered to indicate statistical significance. Baseline characteristics are presented for the whole cohort. Using the Wilcoxon test, individual and total ketones and ratios were compared between participants with or without MASLD. Logistic regression analyses were used to assess the association between circulating FGF21 concentration and circulating ketones with MASLD. Sensitivity analyses were performed to assess this association stratified by sex. Cox proportional hazards models were used to investigate the associations of FGF21 concentration and circulating ketone bodies with all-cause mortality. Sensitivity analyses were performed to assess this association stratified by sex, as well as by cause of death. Due to the skewed distribution, FGF21 and ketone bodies were log_2_-transformed prior to analyses. In logistic and Cox regression analyses, models were adjusted for ketone bodies (analyses of FGF21) and FGF21 (analyses of ketone bodies) and included the interaction term between FGF21 and ketone bodies. Furthermore, the logistic regression and Cox regression models were adjusted for a priori selected potential confounders, including age, sex, waist circumference, eGFR, urinary albumin excretion, smoking status, alcohol intake, total cholesterol, HDL cholesterol, blood pressure, and diabetes. In each model, the interaction term between circulating FGF21 concentration and circulating ketone bodies was included. The effects of interactions were shown using stratified analyses, as well as by plotting the association of each variable with the outcome according to the 10th, 50th, and 90th percentiles of the other variable.

To account for potential bias that could result from the exclusion of participants with missing values, multiple imputations using Fully Conditional Specification were performed using the ‘mice’ package to obtain five imputed data sets. The algorithm was run for 30 iterations, and convergence of the Markov chains was evaluated with trace plots of the mean and variance. Analyses were performed in each of the data sets, and results were pooled using Rubin’s rules [45,46].

To account for better discrimination of the FLI in MASLD, a sensitivity analysis was performed, modifying the cut-off value for case definition in women from ≥60 to ≥32 in logistic regression analyses, adjusted similarly as those described before, and accounting for the interaction term between FGF21 and ketones.

## 5. Conclusions

In this large, population-based cohort, we found that both higher circulating FGF21 concentrations and higher circulating ketone bodies were associated with a higher risk of MASLD, with a positive interaction between these two variables, suggesting that the association between each variable and MASLD intensifies as the levels of the other increases. Similarly, we found that both higher circulating FGF21 concentrations and higher circulating ketone bodies were associated with a higher risk of all-cause mortality, also with a positive interaction between these two variables, suggesting that the association between each variable and all-cause mortality also intensifies as the levels of the other increases. These findings underscore the combined influence of elevated circulating FGF21 concentrations and ketone bodies on the increased risk of MASLD and all-cause mortality, suggesting that these biomarkers may serve as integrated indicators for metabolic risk and potential targets for further investigation.

## Figures and Tables

**Table 1:** Baseline characteristics of the study participants.

Variable	Value
Circulating FGF21 (pg/mL)	888 [534–1374]
Ketone bodies (µmol/L)	176 [139–247]
Age (years)	53 ± 12
Sex (Male, %)	2968 (49)
Waist circumference (cm)	91 [82–100]
eGFR (mL/min/1.73 m^2^)	97 ± 17
Urinary albumin excretion (mg/day)	9 [6–15]
Alcohol intake (>1 drink per day/%)	1558 (26)
Smoking status (n/%)	1689 (28)
Total cholesterol (mmol/L)	5.4 ± 1.0
HDL cholesterol (mmol/L)	1.3 ± 0.3
Systolic blood pressure (mmHg)	126 ± 19
Diabetes (n/%)	324 (5)
Fatty Liver Index	36 [14–67]
MASLD (n/%)	1864 (31)

Data are expressed in median [IQ range] or mean ± SD for continuous variables and in count and percentages for categorical variables. Abbreviations: FGF: fibroblast growth factor; eGFR: glomerular filtration rate; HDL: high-density lipoprotein; MASLD: metabolic-dysfunction-associated steatotic liver disease.

**Table 2:** Logistic regression analyses of circulating FGF21 concentration and ketone bodies with MASLD.

	Circulating FGF21 Concentration		Circulating Ketone Bodies		Interaction Between FGF21 and Ketone Bodies
Model	OR (95% CI)	*p*-Value	OR (95% CI)	*p*-Value	*p*-Value
Model 1	1.93 (1.81–2.05)	<0.001	1.29 (1.19–2.05)	<0.001	0.004
Model 2	1.98 (1.86–2.12)	<0.001	1.21 (1.11–2.12)	<0.001	0.03
Model 3	1.91 (1.79–2.05)	<0.001	1.17 (1.07–1.28)	<0.001	0.03
Model 4	1.82 (1.64–2.02)	<0.001	1.19 (1.03–1.38)	0.01	0.01
Events, n (%)	1864 (31%)		1864 (31%)		

All interaction terms were positive in crude and adjusted models, which suggests that as ketone bodies increase, the odds ratio for the association between FGF21 and MASLD increases, and vice versa. Odds ratios are presented per doubling of the variable of interest. Model 1: Adjusted for ketone bodies (FGF21 analyses) and FGF21 (analyses of ketone bodies), and including the interaction between FGF21 and ketone bodies. Model 2: Model 1 plus adjustment for age and sex. Model 3: Model 2 plus adjustment for creatinine- and cystatin C-based eGFR and urinary albumin excretion. Model 4: Model 3 plus adjustment for waist circumference, alcohol intake, smoking status, total cholesterol, HDL cholesterol, systolic blood pressure, and diabetes.

**Table 3:** Logistic regression analyses of circulating FGF21 concentration and ketone bodies with MASLD stratified across the median values.

**Analyses of FGF21 with MASLD in Participants with Ketone Bodies Below or Above the Median**			
	**Events, n (%)**	**OR (95% CI) per Doubling**	***p*** **-Value**
**Subgroup**			
Ketone bodies < 176 µM	768 (26%)	1.58 (1.35–1.85)	<0.001
Ketone bodies > 176 µM	1096 (36%)	2.05 (1.78–2.37)	<0.001
**Analyses of Ketone Bodies with MASLD in Participants with Ketone Bodies Below or Above the Median**			
	**Events, n (%)**	**OR (95% CI) per Doubling**	***p*** **-Value**
**Subgroup**			
FGF21 < 888 pg/mL	580 (19%)	0.98 (0.78–1.23)	0.86
FGF21 > 888 pg/mL	1283 (43%)	1.41 (1.73–1.70)	<0.001

Analyses are adjusted for age, sex, waist circumference, eGFR, urinary albumin excretion, alcohol intake, smoking status, total cholesterol, HDL cholesterol, systolic blood pressure, and diabetes.

**Table 4:** Cox regression analyses of circulating FGF21 concentration and ketone bodies with all-cause mortality.

	Circulating FGF21 Concentration		Circulating Ketone Bodies		Interaction FGF21 and Ketone Bodies
Model	HR (95% CI)	*p*-Value	HR (95% CI)	*p*-Value	*p*-Value
Model 1	1.24 (1.16–1.32)	<0.001	1.46 (1.34–1.59)	<0.001	0.038
Model 2	1.13 (1.08–1.30)	<0.001	1.18 (1.08–1.30)	<0.001	0.044
Model 3	1.08 (1.01–1.16)	0.029	1.17 (1.06–1.29)	0.001	0.003
Model 4	1.05 (0.97–1.13)	0.17	1.16 (1.06–1.28)	0.002	0.005
Events, n (%)	843 (14%)		843 (14%)		

All interaction terms were positive, which suggests that as ketone bodies increase, the hazard ratio for the association between FGF21 and mortality increases, and vice versa. Hazard ratios are presented per doubling of the variable of interest. Model 1: Adjusted for ketone bodies (FGF21 analyses) and FGF21 (analyses of ketone bodies), and including the interaction between FGF21 and ketone bodies. Model 2: Model 1 plus adjustment for age and sex. Model 3: Model 2 plus adjustment for creatinine- and cystatin C-based eGFR and urinary albumin excretion. Model 4: Model 3 plus adjustment for waist circumference, alcohol intake, smoking status, total cholesterol, HDL cholesterol, systolic blood pressure, and diabetes.

**Table 5:** Cox regression analyses of circulating FGF21 concentration and ketone bodies with all-cause mortality stratified across the median values.

**Analyses of FGF21 with Mortality in Participants with Ketone Bodies Below or Above the Median**			
	**Events, n (%)**	**HR (95% CI) per Doubling**	***p*** **-Value**
**Subgroup**			
Ketone bodies < 176 µM	306 (10%)	1.03 (0.91–1.16)	0.67
Ketone bodies > 176 µM	537 (18%)	1.11 (1.02–1.21)	0.022
**Analyses of Ketone Bodies with Mortality in Participants with Ketone Bodies Below or Above the Median**			
	**Events, n (%)**	**HR (95% CI) per Doubling**	***p*** **-Value**
**Subgroup**			
FGF21 < 888 pg/mL	334 (11%)	1.11 (0.96–1.28)	0.17
FGF21 > 888 pg/mL	508 (17%)	1.26 (1.11–1.43)	<0.001

Analyses are adjusted for age, sex, waist circumference, eGFR, urinary albumin excretion, alcohol intake, smoking status, total cholesterol, HDL cholesterol, systolic blood pressure, and diabetes.

## Data Availability

Data described in the manuscript, code book, and analytic code will be made available upon reasonable request to the editor.

## Supplementary Materials

The following supporting information can be downloaded at https://www.mdpi.com/article/10.3390/ijms26115059/s1.

## Abbreviations

The following abbreviations are used in this manuscript:

AcAc	Acetoacetate
AMPK	AMP-activated protein kinase
BHB	β-Hydroxybutyrate
eGFR	Estimated glomerular filtration rate
FGF	Fibroblast growth factor
FGF21	Fibroblast growth factor 21
FLI	Fatty Liver Index
GFR	Glomerular filtration rate
GGT	Gamma-glutamyltransferase
HDL	High-density lipoprotein
ICD-10	International Classification of Diseases, 10th Revision
IQ	Interquartile
MASLD	Metabolic dysfunction-associated steatotic liver disease
MASH	Metabolic dysfunction-associated steatohepatitis
NMR	Nuclear magnetic resonance
Nrf2	Nuclear factor erythroid 2-related factor 2
OR	Odds ratio
PPARα	Peroxisome proliferator-activated receptor alpha
PREVEND	Prevention of Renal and Vascular End-stage Disease
SD	Standard deviation
T2D	Type 2 diabetes
