# Supplementary material for: Circulating FGF21 and Ketone Bodies Modify the Risk of MASLD and Mortality: Insights from the PREVEND Cohort Study
Source: Int J Mol Sci. 2025 May 24;26(11):5059. doi: 10.3390/ijms26115059 (PMC12154048; doi:10.3390/ijms26115059)
Supplement: Supplementary file 1 [file ijms-26-05059-s001.zip › ijms-3625040-supplementary.pdf]

Supplementary data: Circulating FGF21 and Ketone Bodies Modify the Risk of MASLD and Mortality: Insights from the PREVEND Cohort Study

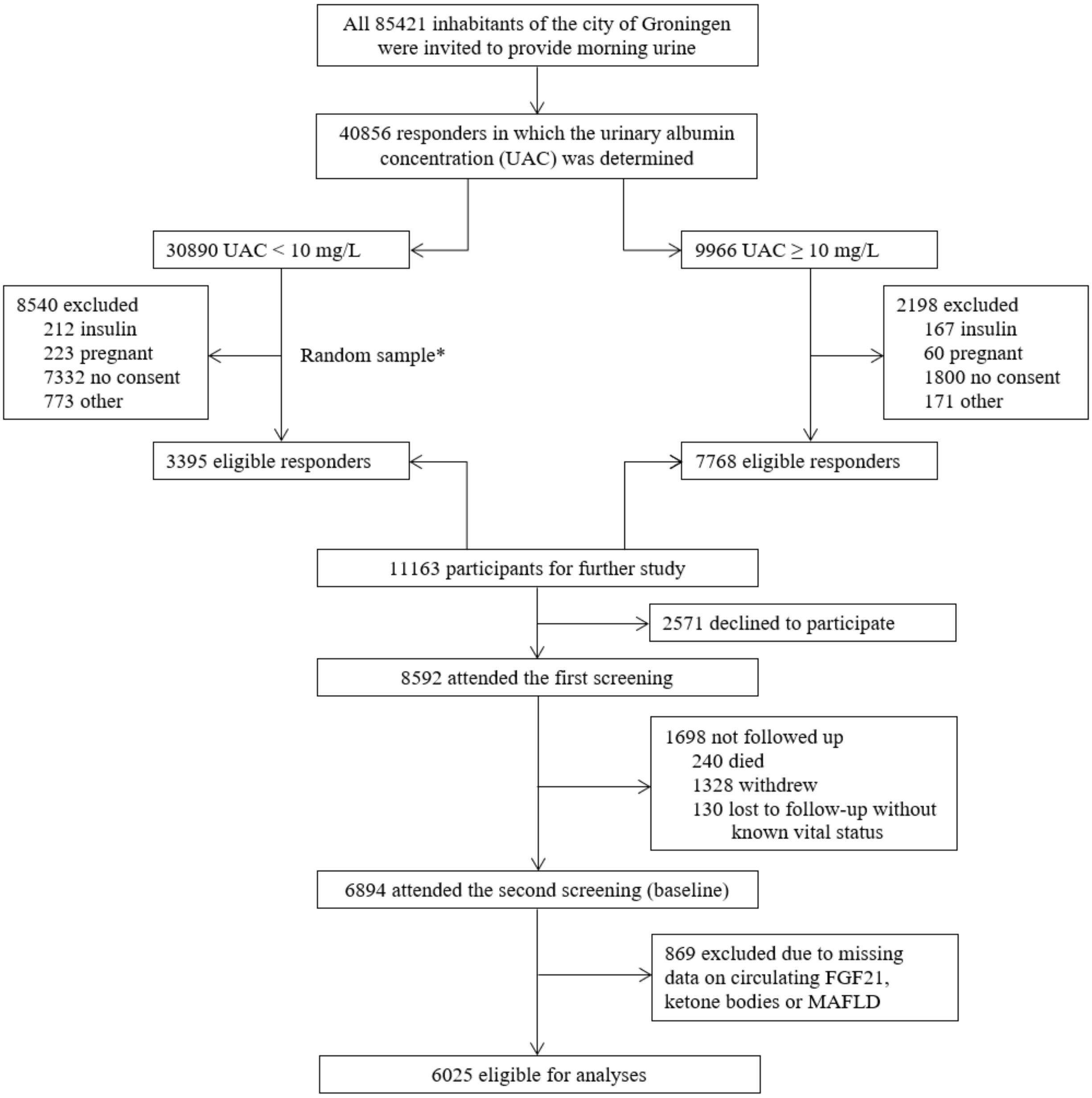

Figure S1. Participant flow-chart

**Table S1.** FGF21, Ketone Bodies and their Ratios in Participants Above or Below a FLI of 60			
	**FLI < 60**	**FLI ≥ 60**	**P-value**
**FGF21, pg/mL**	758.0 [457.5-1194.0]	1188.5 [784.5-1735.0]	<0.001
**Ketone bodies, µmol/L**	168.8 [132.1-241.6]	192.6 [153.0-255.9]	0.01
**BHB, µmol/L**	115.4 [88.0-164.4]	132.1 [103.8-176.9]	<0.001
**AcAc, µmol/L**	37.3 [25.1-56.6]	40.2 [27.5-58.4]	<0.001
**Acetone, µmol/L**	18.8 [12.1-28.1]	21.6 [14.0-31.3]	0.9
**BHB:AcAc**	4.01	3.96	0.2

Data are expressed in median [IQ range] for all variables except BHB:AcAc ratio. FLI: Fatty liver index; FGF: Fibroblast growth factor; BHB: β-hydroxybutyrate; AcAc: Acetoacetate.

**Table S2.** Logistic regression analyses of circulating FGF21 concentration and ketone bodies with MASLD, with different cut-off values of FLI between sexes (Males >60; Females>32).					
	**Circulating FGF21 concentration**		**Circulating ketone bodies**		**Interaction FGF21 and ketone bodies**
**Model**	OR (95% CI)	P-value	OR (95% CI)	P-value	P-value
**Model 1**	2.01 (1.90; 2.13)	<0.001	1.27 (1.18; 1.38)	<0.001	0.01
**Model 2**	1.90 (1.79; 2.01)	<0.001	1.17 (1.08; 1.26)	<0.001	0.02
**Model 3**	1.85 (1.74; 1.96)	<0.001	1.13 (1.04; 1.22)	0.003	0.02
**Model 4**	1.68 (1.53; 1.85)	<0.001	1.10 (0.96; 1.25)	0.2	0.1
**Events, n (%)**	2469 (41%)		2469 (41%)		

All interaction terms were positive in crude and adjusted models, which suggests that as ketone bodies increase, the odds ratio for the association between FGF21 and MASLD increases, and vice-versa.
Odds ratios are presented per doubling of the variable of interest.
Model 1: Adjusted for ketone bodies (FGF21 analyses), FGF21 (analyses of ketone bodies) and including the interaction between FGF21 and ketone bodies.
Model 2: Adjusted for age and sex.
Model 3: Additionally adjusted for creatinine and cystatin C based eGFR and urinary albumin excretion
Model 4: Additionally adjusted for waist circumference, alcohol intake, smoking status, total cholesterol, HDL cholesterol, systolic blood pressure and diabetes.

Table S3. Logistic regression analyses of circulating FGF21 concentration and ketone bodies with MASLD, stratified by sex

	**Circulating FGF21 concentration**		**Circulating ketone bodies**	
**Model**	OR (95% CI)	P-value	OR (95% CI)	P-value
**Model 1**				
Male	1.97 (1.81-2.15)	<0.001	1.26 (1.13-1.40)	<0.001
Female	2.23 (2.01-2.46)	<0.001	1.39 (1.22-1.60)	<0.001
**Model 2**				
Male	1.92 (1.76-2.10)	<0.001	1.17 (1.04-1.31)	0.007
Female	2.05 (1.85-2.27)	<0.001	1.32 (1.15-1.52)	<0.001
**Model 3**				
Male	1.85 (1.70-2.02)	<0.001	1.14 (1.02-1.28)	0.03
Female	1.96 (1.77-2.17)	<0.001	1.27 (1.10-1.47)	<0.001
**Model 4**				
Male	1.98 (1.73-2.26)	<0.001	1.16 (0.96-1.39)	0.1
Female	1.59 (1.33-1.90)	<0.001	1.30 (1.02-1.65)	0.03

Model 1: Adjusted for ketone bodies (FGF21 analyses), FGF21 (analyses of ketone bodies)
Model 2: Adjusted for age.
Model 3: Additionally adjusted for creatinine and cystatin C based eGFR and urinary albumin excretion
Model 4: Additionally adjusted for waist circumference, alcohol intake, smoking status, total cholesterol, HDL cholesterol, systolic blood pressure and diabetes.

Table S4. Logistic regression analyses of circulating FGF21 concentration and ketone bodies with MASLD stratified across the median values, using different FLI cut-off values between sexes (Males >60; Females>32)

Analyses of FGF21 with MASLD in participants with ketone bodies below or above the median.

	**Events, n (%)**	**OR (95% CI) per doubling**	**P-value**
Ketone bodies < 176 uM	1083 (36%)	1.58 (1.37; 1.81)	<0.001
Ketone bodies > 176 uM	1386 (46%)	1.76 (1.55; 2.00)	<0.001

Analyses of ketone bodies with MASLD in participants with ketone bodies below or above the median.

	**Events, n (%)**	**OR (95% CI) per doubling**	**P-value**
FGF21 < 888 pg/mL	817 (27%)	0.93 (0.77; 1.12)	0.4
FGF21 > 888 pg/mL	1651 (55%)	1.27 (1.07; 1.51)	0.008

Analyses are adjusted for age, sex, waist circumference, eGFR, urinary albumin excretion, alcohol intake, smoking status, total cholesterol, HDL cholesterol, systolic blood pressure and diabetes.

Table S5. Logistic regression analyses of circulating FGF21 concentration and ketone bodies with MASLD stratified across the median values.

**Analyses of FGF21 with MASLD in participants with ketone bodies below or above the median value at event occurrence**			
	**Events, n (%)**	**OR (95% CI) per doubling**	**P-value**
**Subgroup**			
Ketone bodies < 193 uM	932 (27%)	1.64 (1.43; 1.90)	<0.001
Ketone bodies > 193 uM	932 (36%)	2.06 (1.76; 2.41)	<0.001
**Analyses of ketone bodies with MASLD in participants with ketone bodies below or above the median value at event occurrence.**			
	**Events, n (%)**	**OR (95% CI) per doubling**	**P-value**
**Subgroup**			
FGF21 < 1190 pg/mL	932 (23%)	1.10 (0.91; 1.32)	0.33
FGF21 > 1190 pg/mL	932 (47%)	1.36 (1.09; 1.71)	0.007

Analyses are adjusted for age, sex, waist circumference, eGFR, urinary albumin excretion, alcohol intake, smoking status, total cholesterol, HDL cholesterol, systolic blood pressure and diabetes.

Table S6. Cox regression analyses of circulating FGF21 concentration and ketone bodies with all-cause mortality, stratified by sex

	**Circulating FGF21 concentration**		**Circulating ketone bodies**	
**Model**	HR (95% CI)	P-value	HR (95% CI)	P-value
**Model 1**				
Male	1.15 (1.06-1.24)	<0.001	1.53 (1.38-1.70)	<0.001
Female	1.44 (1.31-1.59)	<0.001	1.36 (1.17-1.59)	<0.001
**Model 2**				
Male	1.09 (1.01-1.19)	0.04	1.17 (1.04-1.32)	0.008
Female	1.22 (1.08-1.37)	0.001	1.23 (1.04-1.45)	0.01
**Model 3**				
Male	1.04 (0.95-1.14)	0.4	1.17 (1.04-1.31)	0.01
Female	1.16 (1.03-1.31)	0.01	1.20 (1.02-1.42)	0.02
**Model 4**				
Male	1.02 (0.93-1.12)	0.7	1.17 (1.04-1.32)	0.008
Female	1.09 (0.96-1.23)	0.2	1.15 (0.97-1.35)	0.1

Hazard ratios are presented per doubling of the variable of interest.
Model 1: Adjusted for ketone bodies (FGF21 analyses), FGF21 (analyses of ketone bodies)
Model 2: Adjusted for age.
Model 3: Additionally adjusted for creatinine and cystatin C based eGFR and urinary albumin excretion
Model 4: Additionally adjusted for waist circumference, alcohol intake, smoking status, total cholesterol, HDL cholesterol, systolic blood pressure and diabetes.

Table S7. Cox regression analyses of circulating FGF21 concentration and ketone bodies with all-cause mortality, stratified by cause of death (cardiovascular vs. non-cardiovascular)

	**Circulating FGF21 concentration**		**Circulating ketone bodies**	
**Model**	HR (95% CI)	P-value	HR (95% CI)	P-value
**Model 1**				
Cardiovascular	1.21 (1.06-1.38)	0.004	1.67 (1.41-1.97)	<0.001
Non-cardiovascular	1.25 (1.17-1.34)	<0.001	1.39 (1.26-1.54)	<0.001
**Model 2**				
Cardiovascular	1.09 (0.94-1.27)	0.2	1.35 (1.21-1.62)	0.002
Non-cardiovascular	1.15 (1.06-1.24)	<0.001	1.14 (1.02-1.27)	0.02
**Model 3**				
Cardiovascular	1.00 (0.85-1.17)	0.9	1.32 (1.10-1.59)	0.003
Non-cardiovascular	1.11 (1.03-1.20)	0.01	1.13 (1.01-1.26)	0.03
**Model 4**				
Cardiovascular	0.98 (0.83-1.15)	0.8	1.32 (1.10-1.59)	0.004
Non-cardiovascular	1.08 (1.00-1.17)	0.06	1.12 (1.00-1.25)	0.05

Hazard ratios are presented per doubling of the variable of interest.
Model 1: Adjusted for ketone bodies (FGF21 analyses), FGF21 (analyses of ketone bodies)
Model 2: Adjusted for age.
Model 3: Additionally adjusted for creatinine and cystatin C based eGFR and urinary albumin excretion
Model 4: Additionally adjusted for waist circumference, alcohol intake, smoking status, total cholesterol, HDL cholesterol, systolic blood pressure and diabetes.
